# Implication of tau propagation on neurodegeneration in Alzheimer’s disease

**DOI:** 10.3389/fnins.2023.1219299

**Published:** 2023-07-07

**Authors:** Daniel Lamontagne-Kam, Anosha Kiran Ulfat, Vincent Hervé, Tra-My Vu, Jonathan Brouillette

**Affiliations:** Department of Pharmacology and Physiology, Université de Montréal, Montréal, QC, Canada

**Keywords:** Alzheimer’s disease, tau, propagation, neurodegeneration, amyloid, PET, fMRI

## Abstract

Propagation of tau fibrils correlate closely with neurodegeneration and memory deficits seen during the progression of Alzheimer’s disease (AD). Although it is not well-established what drives or attenuates tau spreading, new studies on human brain using positron emission tomography (PET) have shed light on how tau phosphorylation, genetic factors, and the initial epicenter of tau accumulation influence tau accumulation and propagation throughout the brain. Here, we review the latest PET studies performed across the entire AD continuum looking at the impact of amyloid load on tau pathology. We also explore the effects of structural, functional, and proximity connectivity on tau spreading in a stereotypical manner in the brain of AD patients. Since tau propagation can be quite heterogenous between individuals, we then consider how the speed and pattern of propagation are influenced by the starting localization of tau accumulation in connected brain regions. We provide an overview of some genetic variants that were shown to accelerate or slow down tau spreading. Finally, we discuss how phosphorylation of certain tau epitopes affect the spreading of tau fibrils. Since tau pathology is an early event in AD pathogenesis and is one of the best predictors of neurodegeneration and memory impairments, understanding the process by which tau spread from one brain region to another could pave the way to novel therapeutic avenues that are efficient during the early stages of the disease, before neurodegeneration induces permanent brain damage and severe memory loss.

## Introduction

Tau propagation across the brain has been shown to follow a stereotypical pattern in AD using histopathological staining at autopsy more than 30 years ago (Braak and Braak, 1991). It was first reported that fibrillar tau start to accumulate in the trans-entorhinal cortex and then spread to the anterior hippocampus, followed by adjacent temporal and limbic cortex, association isocortex, and ultimately to primary sensory cortex (Braak and Braak, 1991; Braak and Del Tredici, 2015; Cho et al., 2016).

Novel technologies allowing for the first time the visualization and quantification of aggregated, paired helical filament (PHF) tau in the brain of living people using positron emission tomography (PET) (Moscoso et al., 2022). Early increase in tau PET uptake was validated in the entorhinal cortex but was also found in many other regions such as the inferior temporal lobe, amygdala, banks of the superior temporal sulcus, fusiform gyrus, inferior parietal lobe, middle temporal lobe and the precuneus (Insel et al., 2020, 2023). Using novel 3D neuroimaging techniques, the noradrenergic locus coeruleus has also been shown as a very early accumulation site for trans-neuronal spreading of hyperphosphorylated tau (Gilvesy et al., 2022). Moreover, there is a growing body of evidence suggesting that substantial inter-individual variabilities in the pattern and intensity of tau signal may be more common than previously expected in affected AD brain regions (Murray et al., 2011; Scholl et al., 2017; Hanseeuw et al., 2019; Jack et al., 2019; Lowe et al., 2019; Betthauser et al., 2020; Vogel et al., 2021).

**Figure 1:**
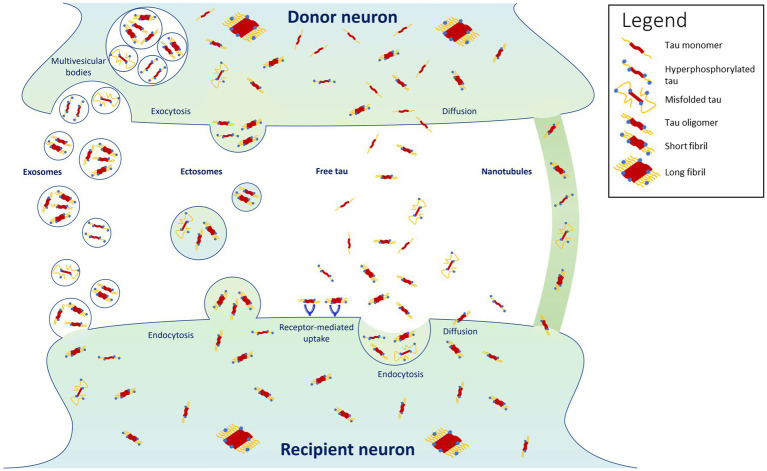
Proposed mechanisms for tau propagation. Highlighted are the findings that tau propagates predominantly in its oligomeric and short-fibril forms, with misfolded and hyperphosphorylated forms propagating in smaller proportions (Colom-Cadena et al., 2023). Tau monomers and long fibrils do not appear to propagate from cell to cell (Wu et al., 2013).

It is now widely recognized that tau can propagate, at least partly, by being secreted in an activity-dependent manner into the synaptic cleft from donor pre-synaptic neurons, and then recapture by recipient post-synaptic neurons localized in another brain region (Mohamed et al., 2013; Pooler et al., 2013; Calafate et al., 2015; Wu et al., 2016; Mudher et al., 2017; Vogel et al., 2020) (Figure 1). Tau can also be released as a result of leakage from neurodegeneration of the pre-synaptic neuron and diffuse in its close environment. Using high-resolution array tomography on post-mortem temporal and occipital cortices of AD patients, it was found recently that phosphorylated or misfolded tau, but mostly oligomeric tau accumulates in both pre- and post-synaptic terminals of the same synapses (Colom-Cadena et al., 2023), suggesting that oligomeric tau could be the main species of tau that spreads trans-synaptically. These results are in accordance with another *in vitro* study showing that low molecular weight tau aggregates and short fibrils (but not monomers, long fibrils, nor long tau filaments), are internalized through endocytosis and transported anterogradely and retrogradely (Wu et al., 2013). Moreover, trans-synaptic tau spreading has also been shown in various animal models overexpressing human tau or using adeno-associated virus-mediate expression of tau (de Calignon et al., 2012; Harris et al., 2012; Liu et al., 2012; Pickett et al., 2017; Wegmann et al., 2019).

Propagation of tau pathology is one of the strongest predictors of progressive neurodegeneration and cognitive decline in AD (La Joie et al., 2020; Biel et al., 2021; Ossenkoppele et al., 2021b). Furthermore, elevated tau burden has been strongly associated with higher risk of progression from a clinically unimpaired to a mild cognitive impairment (MCI) status (Strikwerda-Brown et al., 2022). Thus, determining the factors that influence tau accumulation and propagation could help develop new treatments to prevent or halt the neurodegenerative process and ensuing cognitive decline that takes place in the early stages of AD. Here, we will review the latest research performed in the field of tau propagation in the human brain using neuroimaging techniques such as PET, magnetoencephalography (MEG), and functional magnetic resonance imaging (fMRI).

### Impact of amyloid-beta (Aꞵ) on tau propagation

Many PET studies have consistently emphasized the critical role of Aꞵ pathology on the accumulation and spreading of tau (Wang et al., 2016; Jacobs et al., 2018; Franzmeier et al., 2020; Vogel et al., 2020; Jiang et al., 2022; Lee et al., 2022). Since Aꞵ starts to accumulate before tau in the preclinical stage of AD (Sperling et al., 2011), it is often difficult to disentangle the phenomena that are solely and specifically attributed to tau pathology. Although the interaction between Aꞵ and tau still needs to be fully determined, many studies have shown that Aꞵ greatly contribute to the deleterious effects of tau in AD brain. It was found that AD patients and cognitively normal people who tested positive for Aꞵ_42_ into the cerebrospinal fluid (CSF) had higher tau PET signal in the cortex and neurodegeneration in the hippocampus (Wang et al., 2016).

Higher neocortical accumulation of Aꞵ also predicted hippocampal volume loss, abnormalities in the white-matter tract that projects from the hippocampus to the posterior cingulate cortex (PCC), larger tau deposition in PCC, and faster memory decline in healthy older individuals (Jacobs et al., 2018). Other studies also found that detection of higher amyloid PET signal also extended tau PET uptake in cortical brain regions beyond the entorhinal cortex and correlates with cognitive decline (Jack et al., 2018; Pontecorvo et al., 2019; Sanchez et al., 2021). These findings in humans are consistent with animal studies showing that Aꞵ is an instigator of trans-synaptic spread of tau across the brain (de Calignon et al., 2012; Ahmed et al., 2014; Pooler et al., 2015; He et al., 2018).

Using an epidemic spreading model of tau along the AD continuum, Vogel and colleagues found that brain areas with higher Aβ burden had more tau accumulation than predicted by connectivity patterns (Vogel et al., 2020). This role of Aβ in accelerating tau spread was also observed in another study showing that individuals without amyloid plaque had almost no tangles in their brain, whereas those with Aꞵ PET uptake had more tangles at baseline and during follow-up trials (Franzmeier et al., 2020). Moreover, Aꞵ was found to interact with tau within the inferior temporal gyrus and accelerate widespread neocortical tau propagation (Lee et al., 2022). Altogether, these data indicate that temporal and spatial patterns of tau pathology depend on prior Aꞵ deposition. Although the exact mechanisms by which Aꞵ affects the accumulation and propagation of tau in distant brain regions still need to be elucidated, novel PET studies in human are giving new insights on how Aꞵ and tau pathologies are linked and influence each other in AD pathogenesis.

### Effect of structural, functional, and proximity connectivity on tau spreading

Three models have been proposed as predictors for tau propagation. The functional connectivity model suggests that tau propagation is more likely to be detected in connected brain regions that fire together, whereas the structural and proximity connectivity models, respectively, propose that tau propagation relies more on direct synaptic connections or physical distance between brain regions (Schoonhoven et al., 2022).

Many cross-sectional studies have reported that the level of tau accumulation strongly correlates among regions that are functionally connected (Hoenig et al., 2018; Jacobs et al., 2018; Franzmeier et al., 2019b; Vogel et al., 2020). By combining magnetoencephalography (MEG) to measure electrical activity in the brain with positron emission tomography (PET) to evaluate tau depositions at several stages of AD, a recent study by Schoonhoven and colleagues has shown that the functional connectivity model was most accurate (*r* = 0.58) compared to the structural (*r* = 0.45) and proximity model (*r* = 0.44) at predicting tau propagation in the preclinical stage of AD (Schoonhoven et al., 2022). Since the prediction accuracy of the functional networks declined with AD progression, this suggests that exacerbated neuronal communication might be especially important for tau spreading in the earlier stages of the disease, before initial neuronal hyperactivity progressively switch to hypoactivity in AD (Busche and Konnerth, 2016; Hector and Brouillette, 2020).

In another study using resting-state fMRI scans to evaluate connectivity between 400 brain regions throughout the AD continuum, a strong correlation was found between functional connectivity and an increase in tangles, whereas spatial proximity was not a robust predictor of tau accumulation unless the nearby brain areas were functionally connected (Franzmeier et al., 2020). Connectivity, rather than proximity, was also identified as the primary source of tau spreading in an *in vivo* model of tau propagation using human P301S tau transgenic mice injected with brain extract containing tau aggregates (Ahmed et al., 2014).

These results are in line with an *in vivo* microdialysis study showing that higher neuronal activity induced by presynaptic glutamate release increased the level of extracellular tau in the hippocampus of wild-type mice (Yamada et al., 2014). Moreover, it was reported that higher neuronal activity induced by glutamate, (*S*)-AMPA or KCl depolarization increased tau release from primary mature cortical cultures, whereas inhibition of neuronal activity with tetrodotoxin impairs AMPA-mediated tau release (Pooler et al., 2013).

Although synaptic activity has been established as an important predictor of tau propagation, other studies have also highlighted the importance of the structural connectivity model to determine the pattern of tangles within the brain. In a study using resting-state fMRI, DTI, and tau PET, it was reported that anatomical connections could predict 70% of the tau PET pattern observed in the brain of MCI and AD patients, whereas the functional and proximity connectivity models, respectively, explained 58 and 48% of the tangle pattern (Vogel et al., 2020). Even though it is not clear at the moment if methodological differences between this study and others like the one by Schoonhoven et al. can explain the primary role for structural over functional connections, it seems nonetheless that both models influence tau propagation in a way that is consistent with the idea that tau spread through long-range axonal connections.

The observation that tau can propagate in both retrograde and anterograde directions of neural networks also argue in favor of an anatomical propagation of tau (Ahmed et al., 2014; Takeda et al., 2015), independent of neuronal activity that occurs unilaterally from the pre- to the post-synaptic neurons. However, it cannot be excluded that at least part of the retrograde pattern of tau propagation could be the result of residual tau that was released during synaptic activity and then taken up retroactively.

In addition to this cell-to-cell tau transmission, simple diffusion of tau release by neurons might also explain its local spread. Given that the clearing rate of tau from the extracellular space is relatively slow with a half-life of about 11 days (Yamada et al., 2014), this favors its diffusion into the interstitial fluid (ISF) within nearby brain regions. This is even more marked when neurodegeneration becomes more prominent in the late phase of the disease, when higher levels of tau leak from dying cells, and breakdowns in the glymphatic and immune system slow tau clearance (Haage and De Jager, 2022; Ishida et al., 2022). Altogether, these results suggest that tau spreading in a stereotypical manner in the brain of AD patients depends on trans-synaptic propagation especially among active neurons that fire together, and that local diffusion of tau also participate in the dispersion pattern of tau that closely correlates with progressive neurodegeneration and memory impairment seen during AD pathogenesis.

### Role of starting localization of tau accumulation

Another aspect that can dictate the speed and pattern of tau propagation is the brain regions where tau starts to accumulate during AD. Although tau pathology spreads in a stereotypical pattern in AD (Braak and Braak, 1991), many studies have reported substantial individual variations in tau PET pattern, particularly in preclinical AD (Murray et al., 2011; Scholl et al., 2017; Ossenkoppele et al., 2020; Vogel et al., 2020; La Joie et al., 2021; Vogel et al., 2021; Frontzkowski et al., 2022; Young et al., 2022). By combining tau PET scans with resting-state fMRI to map brain connectivity, it was reported that tau spreads more rapidly when it is located in highly connected hub regions of the fronto-parietal association cortex compared to less connected regions in the temporo-limbic and visual cortices (Frontzkowski et al., 2022). Interestingly, AD patients with symptoms at a younger age were more likely to have tau deposition in hub regions, while participants who developed symptomatic AD at an older age had more tangles in limbic areas (Frontzkowski et al., 2022). Since hubs in the fronto-parietal network are essential for complex cognitive function (Cole et al., 2013; Zanto and Gazzaley, 2013), this could explain why stronger tau pathology in these hub regions was also associated with faster cognitive decline (Frontzkowski et al., 2022).

**Figure 2:**
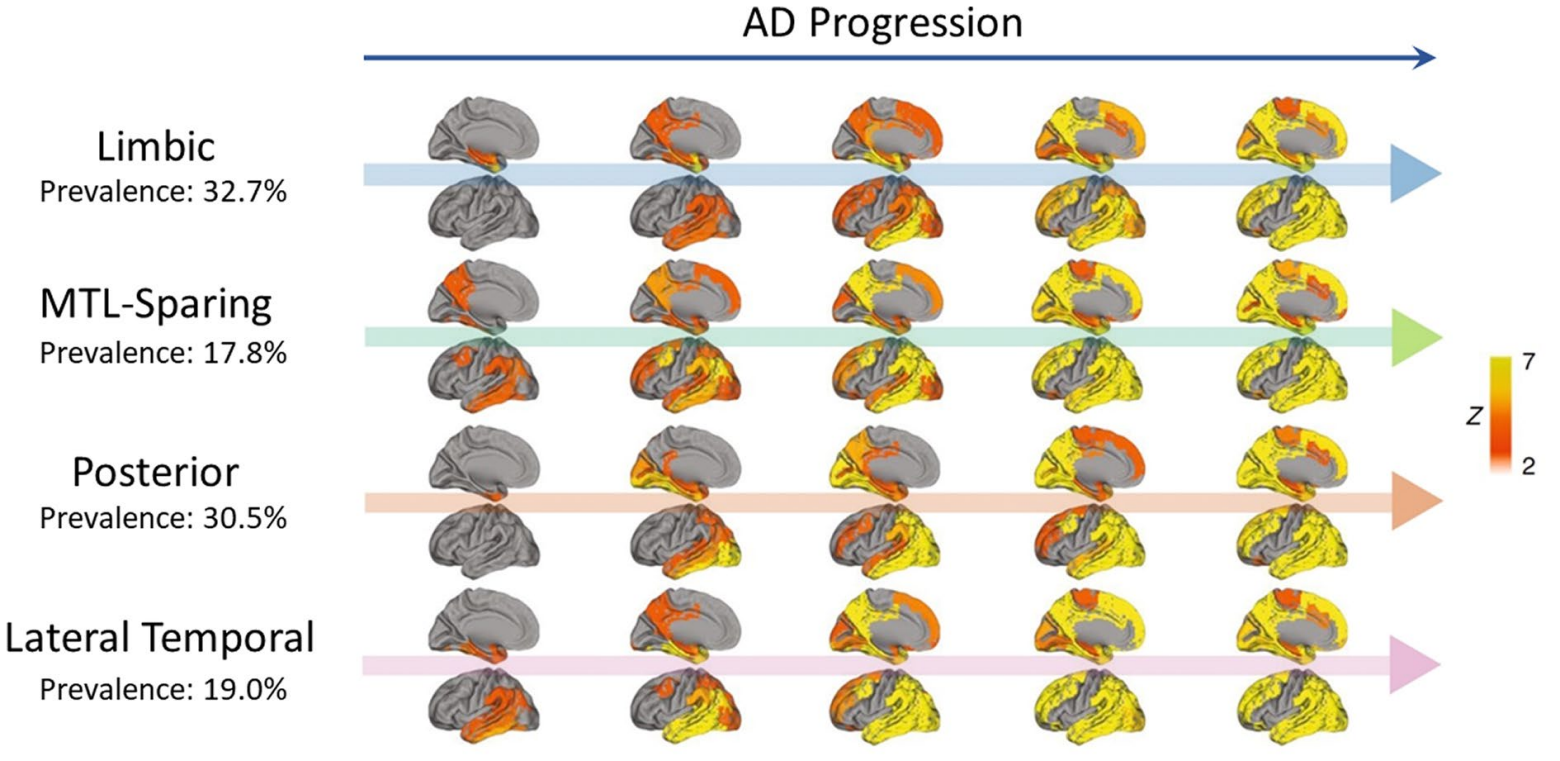
Representation of four distinct tau propagation patterns as determined by PET in a cross-sectional analysis (*n* = 2,324) (Vogel et al., 2021), with AD progression presented from left to right. The prevalence of each pattern in the analysis is indicated below the pattern names. Only the left side of the brain is shown, with the interior faces of sagittal cuts above their respective arrows. Different points of origin are highlighted in each of the defined patterns. Limbic propagation most closely resembles patterns reported by Braak and Braak (1991). Figure adapted from Vogel et al. (2021).

Different patterns of tau propagation were also observed in different subtypes of AD. Using PET scans from 1,612 participants covering the full clinical AD spectrum, tau was found to spread in four distinct spatiotemporal trajectories, including the limbic-predominant and medial temporal lobe (MTL)-sparing patterns as well as the posterior and lateral temporal patterns (Murray et al., 2011; Whitwell et al., 2012; Ferreira et al., 2020; Ossenkoppele et al., 2020; Vogel et al., 2021) (Figure 2). In early-onset AD (< 65 years), tau also tended to accumulate more in the prefrontal, premotor, and inferior parietal cortices than in late-onset AD (Scholl et al., 2017). Moreover, systematic spatiotemporal variations in tau spreading that deviate from the Braak staging system have been observed in clinical variants of AD, such as posterior cortical atrophy and logopenic primary progressive aphasia (Gorno-Tempini et al., 2011; Ossenkoppele et al., 2016; Crutch et al., 2017).

Tangles were also found to spread asymmetrically *in vivo*, with more tau pathology in the right entorhinal cortex for most participants (Vogel et al., 2020). This right-side epicenter was associated with more tau-tracer uptake on PET scans in frontal region and was mainly observed in older AD patients. Although we do not know at the moment why tau accumulation begins in distinct brain areas and follows variable spatiotemporal patterns in different people, it will be important to incorporate this heterogeneity into tau spreading models to hopefully one day be able to predict tau propagation at the individual level. Since tau pathology is the key driver of neurodegeneration and cognitive decline in AD (La Joie et al., 2020; Biel et al., 2021; Ossenkoppele et al., 2021b), understanding the cellular and molecular mechanisms underlying the different spatial patterns of tau distribution that sustained the diverse clinical manifestation of the disease could pave the way to develop new therapies that prevent cell death and memory deficits.

### Genetic variants involved in tau propagation

Genetic variants could be, at least partly, a factor underpinning the variability of tangle spread in AD. Genome wide-association studies (GWAS) performed over the last few decades have underlined several genes such as APOE and BIN1 that increase the probability of developing AD (Jansen et al., 2019; Wightman et al., 2021). Although it is well-established that APOE4 is the strongest genetic predictor of sporadic AD and is associated with early amyloid deposition rate and burden (Corder et al., 1993; Ossenkoppele et al., 2015; Ridge et al., 2016; Lim et al., 2017; Ge et al., 2018; Mishra et al., 2018; Toledo et al., 2019; Insel et al., 2021), a growing body of evidences suggests that APOE4 is also involved in tau pathology (Table 1).

**Table 1:** The main genetic factors involved in the propagation of tau in AD.

Gene name	Abbreviation	Functions	Impact on tau	References
Apolipoprotein E	APOE	- Lipid transport, metabolism, and homeostasis - Innate and adaptive immune responses - A major genetic factor for sporadic AD	- APOE4 directly increased tau PET burden in medial temporal lobe beyond effects attributable to amyloid accumulation - APOE4 increased tau accumulation and reduced its clearance - APOE2 was associated directly with reduced tau accumulation	Lin et al. (2018) , Young et al. (2023), Shi et al. (2017), and Mentis et al. (2021)
Bridging integrator 1	BIN1	- Organization and control of myelination - Major AD risk variant found in many GWAS	- Increase probability of developing AD by modulating tau toxicity - BIN1 isoform is reduced in AD brain and a lower level of BIN1 is known to induce tau propagation in cultured neurons	Calafate et al. (2015), Jansen et al. (2019), and Wightman et al. (2021)
Clusterin	CLU	- Involved in lipid transport and is released in response to cell stress	- Upregulation of Clusterin can enhance tau seeding and possibly accelerate the spreading of tau pathology	Yuste-checa et al. (2021)
Klotho	KL	- Regulation of oxidative stress, growth factor signaling, and ion homeostasis	- A variant of KL (KL-*VS* heterozygosity) was correlated with lower tau accumulation	Neitzel et al. (2021)
Phosphatidylinositol binding clathrin assembly protein	PICALM	- Involved in clathrin-mediated endocytosis, regulates APP internalization and subsequent Aβ generation	- PICALM co-localizes with tau inclusion in AD and other tauopathies	Ando et al. (2013, 2016)
Protein tyrosine kinase 2 beta	PTK2B	- Involved in a cell adhesion pathway	- PTK2B was identified as a modulator of tau pathology	Dourlen et al. (2017)
Triggering receptor expressed on myeloid cells 2	TREM2	- Involve in immune response and chronic inflammation - Role in microglial proliferation, survival, clustering, and phagocytosis	- The R47H variant of TREM2 increased the level of total tau protein in the CSF without affecting Aβ42	Guerreiro et al. (2013) and Lill et al. (2015)

In a study using preclinical AD individuals that are cognitively healthy but have abnormal amyloid level, it was found that APOE4 directly increased tau PET burden in medial temporal lobe (entorhinal cortex and amygdala) beyond effects attributable to amyloid accumulation (Young et al., 2023). APOE4 also had a deleterious influence on tau deposition in the early neocortical regions (inferior temporal, inferior parietal, precuneus), but these effects were mostly mediated by amyloid load. Conversely, APOE2 was associated directly with reduced tau accumulation in medial temporal lobe and early neocortical regions, which highlights APOE2 as a key protective variant (Young et al., 2023).

These results are consistent with previous PET reports showing that APOE4 carriers across the entire AD continuum have a more medial temporal lobe-dominant pattern of tau burden even after controlling for amyloid accumulation (Livingston et al., 2017; Mattsson et al., 2018; Therriault et al., 2020; La Joie et al., 2021; Ossenkoppele et al., 2021a). These findings are also in line with studies performed in iPSC-derived human brain cells and mouse models showing that APOE4 worsens tau deposition and neurodegeneration (Shi et al., 2017; Lin et al., 2018; Wang et al., 2018), and may have a role in reducing tau clearance through meningeal lymphosclerosis (Mentis et al., 2021; Ishida et al., 2022). However, it should be noted that other reports have not observed a significant impact of APOE4 on tau burden in clinically unimpaired individuals after adjusting for amyloid load (Lowe et al., 2018; Ramanan et al., 2019). These contrasting observations showcase that APOE4 may be an underlying factor for the accumulation of tau but not the only factor.

Another AD risk variant found in many GWAS is the gene BIN1 (i.e., bridging integrator 1), which encodes for a nucleoplasmic adaptor protein involved in many processes such as the regulation of neuronal excitability (Voskobiynyk et al., 2020), presynaptic vesicle release (De Rossi et al., 2020), and clathrin-mediated endocytosis (Calafate et al., 2016; Crotti et al., 2019). Higher gene expression of BIN1 found in AD brain has been found to increase trans-neuronal tau spreading (Chapuis et al., 2013; Calafate et al., 2016; Crotti et al., 2019), and to be associated with more pronounced tau pathology but not higher Aꞵ burden (Holler et al., 2014; Taga et al., 2020). Moreover, BIN1 rs744373 risk allele carriers were reported to have a higher tau PET signal, faster cognitive decline, and accelerated tau PET accumulation rate when Aꞵ is found at higher levels (Franzmeier et al., 2019a, 2022). Conversely, a protective variant of the klotho gene (KL-VS^het^) that occurs in 20–25% of the population (Dubal et al., 2014), was correlated with lower amyloid-associated tau accumulation (Neitzel et al., 2021), better cognitive performance (Deary et al., 2005; Dubal et al., 2014; Neitzel et al., 2021), and longer life expectancy (Arking et al., 2005; de Vries et al., 2017).

To have a broader view of the impact of genetic risk factors on tau pathology, Rubinski and colleagues have generated a polygenic score (PGS) from 85 GWAS single-nucleotide polymorphisms SNPs linked to AD, excluding APOE (Rubinski et al., 2023). They observed that an elevated PGS was associated with a faster rate of fibrillar tau progression and cognitive impairment, especially when amyloid plaque levels were more intense. Altogether, these results indicate that many gene risk factors have additional influences on the progression of tau pathology. Investigating the cellular and molecular pathways underpinning these effects could lead to novel therapeutic strategies to at least delay tau-dependent neurodegeneration and cognitive deficits in AD.

### Tau hyperphosphorylation and spreading

Human tau protein has 85 potential phosphorylation sites, 9 of which are phosphorylated in nonpathological, soluble tau, and 45 of which have been identified as being phosphorylated in insoluble tau from AD patients (Hanger et al., 2007). The levels of phosphorylation at certain epitopes of tau also seems to have an impact on the spreading of tau fibrils. In a recent study by Pichet Binette and colleagues, it was observed that amyloid-related increases in soluble phosphorylated tau (p-tau) at epitope 217 in CSF was associated with cognitive decline and faster accumulation of tau aggregates in the early stages of AD, especially in regions that are functionally connected to areas where tau pathology started (Pichet Binette et al., 2022). However, when amyloid plaques and soluble p-tau had plateaued in later stages of the disease, soluble p-tau217 lost its effect on tangle accumulation, and cognitive deficits were more associated with the accumulation rate of insoluble tau aggregates. Thus, these results suggest that targeting soluble p-tau could be an interesting therapeutics strategy in early AD to prevent or slow down cognitive decline, the formation of insoluble tau aggregates and ensuing neurodegeneration.

To determine how amyloid plaques increase p-tau levels in the early stages of AD, Biel and colleagues (Biel et al., 2023) evaluated if tau phosphorylation was affected by the soluble fragment of triggering receptor expressed on myeloid cell 2 (sTREM2), a microglial activation marker increased in the CSF of mild AD patients that also correlates with CSF tau (Heslegrave et al., 2016; Piccio et al., 2016). They found that higher level of fibrillar Aꞵ was associated with increased CSF concentrations of sTREM2, and that sTREM2 mediated the association between fibrillar Aꞵ and the increase of CSF p-tau181 in early Aꞵ-accumulator individuals, which have high levels of Aꞵ_1-42_ in the CSF but subthreshold levels of Aꞵ when measured by PET. Conversely, in late Aꞵ-accumulators with high levels of Aꞵ found in both CSF and PET, higher level of sTREM2 was no longer correlated with fibrillar Aꞵ but paralleled CSF p-tau181 increases, indicating that sTREM2 is more tightly linked to soluble p-tau181 when high amounts of Aꞵ fibrils are detected in the brain.

These results are in line with a previous study showing that sTREM2 levels correlated with total and phosphorylated tau in the CSF in dominantly inherited AD approximately 5 years before the onset of symptoms (Suarez-Calvet et al., 2016). It also agrees with another report showing that amyloid-associated microglial activation correlates with both tau pathology and cognitive decline (Pascoal et al., 2021). Since plasma p-tau231 has been shown recently to be the earliest marker of amyloid aggregation (Mila-Aloma et al., 2022), it will be interesting to determine if p-tau231 affects tau phosphorylation and propagation across the entire AD spectrum.

## Conclusion

Neuroimaging techniques indicate that the connectomic architecture of the brain dictates tau accumulation and spreading, which are critical for neurodegeneration and memory decline that begin in the early stages of AD. PET studies also highlight the close relationship between Aꞵ and tau, and how they drive neuropathological phenotypes observed in the entire AD continuum. Further studies are needed to decipher why tangle propagation is so heterogenous between brain regions and individuals. Finding protective factors that prevent or decrease the rate of tau deposition and spreading could also pave the way to develop therapeutic strategies against neurodegeneration and cognitive impairment in AD.

## Author contributions

DL-K, AKU, VH, T-MV, and JB wrote, revised, and edited the manuscript. All authors contributed to the article and approved the submitted version.

## Conflict of interest

The authors declare that the research was conducted in the absence of any commercial or financial relationships that could be construed as a potential conflict of interest.

## Publisher’s note

All claims expressed in this article are solely those of the authors and do not necessarily represent those of their affiliated organizations, or those of the publisher, the editors and the reviewers. Any product that may be evaluated in this article, or claim that may be made by its manufacturer, is not guaranteed or endorsed by the publisher.
